# Complex Health Needs in Hurricane-Affected Youth and Their Families: Barriers, Vulnerabilities, and Mental Health Outcomes

**DOI:** 10.1007/s10802-024-01279-6

**Published:** 2025-01-22

**Authors:** Jessica T. Kelly, BreAnne A. Danzi

**Affiliations:** Department of Psychology, University of South Dakota, 414 East Clark Street, Vermillion, SD USA

**Keywords:** Complex Health Needs, Youth, Disaster, Posttraumatic Stress, Depression, Anxiety

## Abstract

Youth with complex health needs (CHNs; e.g., requiring daily assistance or equipment for care) and their parents face heightened vulnerabilities during natural disasters, potentially leading to poorer mental health outcomes compared to those without CHNs. However, limited research has focused on this group’s disaster-related experiences and their impact on mental health outcomes. This study aimed to investigate the disaster experiences, perceptions, and mental health outcomes of youth with CHNs and their parents’ post-hurricane and to evaluate the unique influence of CHN- and disaster-related factors on their psychological functioning. Parents (*N* = 142) of youth with CHNs (*n* = 48) and without CHNs (*n* = 94) who experienced a hurricane reported on their youth’s and their own psychological functioning, disaster perceptions, experiences, and CHN-related information. Youth with CHNs exhibited greater perceived life threat compared to youth without CHNs. Families of youth with CHNs were more likely to evacuate and faced greater evacuation barriers. They also exhibited greater PTS, depressive, and anxiety symptom severity compared to those without CHNs. Financial healthcare concerns were not associated with youth with CHNs or their parents’ mental health symptomatology. Hurricane-impeded access to healthcare necessities was associated with youth and parent PTS and depressive symptom severity and youth anxiety symptom severity. These findings underscore the vulnerabilities of youth with CHNs and their parents’ post-hurricane, emphasizing the need for tailored mental health services and improved disaster planning resources to support this population effectively.

Approximately three million youth in the United States and 220 million internationally live with complex health needs (CHN; Young, 2021; United Nations Department of Economic and Social Affairs, n.d.). CHNs refer to any medical (e.g., epilepsy, diabetes), behavioral (e.g., anxiety, depression), developmental (e.g., congenital heart defects, spina bifida), or other health condition that requires additional assistance with daily functioning or reliance on medical equipment for health maintenance (Individuals with Disabilities Education Act, 2004; Centers for Disease Control and Prevention, 2020). The unique combination of CHN- and disaster-related factors present for these groups are unique vulnerabilities that place youth with CHNs and their families at heightened risk for poor psychological outcomes following disasters (Peek & Stough, 2010; Ducy & Stough, 2021; Stough et al., 2017). Despite extensive identification, these vulnerabilities are largely unconsidered in crucial disaster information, often complicating disaster response and threatening their well-being (Peek & Stough, 2010). Indeed, little is known regarding how these unique experiences impact their psychological functioning. The purpose of this study was to understand the hurricane-related experiences of youth with CHNs and their families, how these experiences impacted their psychological functioning, and how such functioning differed between youth with CHNs and their families and those without CHNs.

## Vulnerabilities of Youth with CHNs and Their Parents

The daily experiences of youth and their families are highly impacted by CHNs. Specifically, youth with CHNs and their parents may experience stigma that complicates access to functional resources and contributes to financial stress (Goudie et al., 2014; Werner & Shulman, 2013). Youth with CHNs have been found to experience greater mental health difficulties compared to youth without CHNs (Berg et al., 2015), and parents of youth with CHNs have been found to experience greater stress, lower quality of life, and greater anxiety-related symptoms compared to parents of youth without CHNs (Goudie et al., 2014; Werner & Shulman, 2013).

### Disaster-Driven Vulnerabilities Before, During, and After Disasters

Beyond the vulnerability of youth during natural disasters, youth with CHNs experience myriad unique vulnerabilities before, during, and after disasters. During evacuation, youth with CHNs are at an increased risk of exposure to danger and injury due to a limited self-defense capacity, separation from caregivers, impeded access to healthcare necessities (i.e., durable medical equipment), and evacuation barriers such as little access to transportation or accessible shelters (Peek & Stough, 2010; Ducy & Stough, 2021; Stough et al., 2017). Youth with CHNs and their families are less likely to evacuate compared to those without CHNs (Peek & Stough, 2010). After evacuation, physical and mental health difficulties may worsen due to environmental debris, limited access to medical resources, and CHN-informed mental healthcare (Stough et al., 2017; Ducy & Stough, 2021). Further, this group often experiences reduced access to information supporting resiliency and recovery (Peek & Stough, 2010). Lastly, youth with CHNs often experience long-term educational disruptions, which require tailored plans and resources that are less available in the aftermath of disasters.

Given these unique vulnerabilities, perceived disaster threat may present differently among youth with CHNs. Specifically, *perceived life threat*, or whether youth perceive their lives are in danger beyond actual life threat, may be heightened in youth with CHNs compared to youth without CHNs. Notably, perceived life threat may be a better predictor of post-disaster functioning in youth beyond actual threat (McDonald et al., 2019). Given such medical difficulties, it may be that the heightened physical vulnerability and uncertainty related to access to critical lifesaving and sustaining equipment and caretakers may influence perceptions of threat to life in this sample, subsequently leading to poor mental health outcomes following disasters. However, it is presently believed that no study has evaluated perceived life threat in youth with CHNs. The present study seeks to further characterize the unique vulnerabilities and barriers experienced by youth with CHNs while evaluating parent reports of disaster perceptions held by this group that likely have significant implications for resiliency.

## Mental Health Outcomes of Youth with CHNs and Their Parents Following Disasters

### Youth Mental Health

Youth with CHNs exhibit adverse reactions emotional and behavioral reactions to trauma, much like other youth without CHNs (Sorour et al., 2014). In fact, given the myriad medical vulnerabilities experienced by youth with CHNs, it may be that youth with CHNs, and their parents are at risk for adverse reactions to natural disasters. However, little research has specifically evaluated the mental health functioning of this population following natural disasters. To the best of our knowledge, only a handful of studies have evaluated mental health functioning in youth with CHNs following natural disasters.

Following natural disasters, current literature has found that youth with CHNs exhibit PTS (Rath et al., 2007; Takada, 2013; Ducy & Stough, 2021; Mehtar & Mukaddes, 2011), depressive (Rath et al., 2007), anxiety (Rath et al., 2007), and behavioral (Rath et al., 2007, Takada, 2013; Mehtar & Mukaddes, 2011) reactions to trauma, in addition to lower quality of life (Davis et al., 2023). In fact, youth with CHNs were found to exhibit greater PTS, depressive, anxiety, and behavioral symptomatology when compared with those of youth without CHNs in one sample (Rath et al., 2007). Youth with CHNs were more likely than youth without CHNs to live in disaster-damaged homes (Rath et al., 2007). Additionally, youth with CHNs and their families were identified as experiencing a variety of CHN-related vulnerabilities complicated by the nature of the disaster including worsened health (Rath et al., 2007), impacted access to healthcare (i.e., medical doctor visits and limited access to medication; Rath et al., 2007; Ducy & Stough, 2021; Davis et al., 2023), inaccessible shelters (Takada, 2013), and difficulty receiving accessible information and services tailored to enhancing recovery and resiliency in youth with CHNs (i.e., lack of information provided to parents regarding the unique ways in which youth with CHNs exposed to disasters respond to trauma; Ducy & Stough, 2021). In fact, such vulnerabilities faced by youth with CHNs were observed to further exacerbate mental health among these youth, specifically heightened anxiety symptoms and lower quality of life (Takada, 2013; Davis et al., 2023).

While these studies offer important insights into the vulnerabilities of youth with CHNs exposed to disasters, the generalizability of such findings is limited by generalizability barriers due to qualitative methodologies (e.g., Takada, 2013; Ducy & Stough, 2021), unstandardized assessment of youth mental health symptoms (e.g., Takada, 2013; Ducy & Stough, 2021), generalizability across different types of CHNs (e.g., Ducy & Stough, 2021; Davis et al., 2023; Mehtar & Mukaddes, 2011), and limited assessment of youth mental health outcomes (e.g., Davis et al., 2023). The current study will add to the current understanding of the mental health consequences and disaster-related experiences of youth with CHNs while also utilizing structured, quantitative methodologies among youth with diverse CHNs and CHN-related experiences. Moreover, the current study is novel in its approach to evaluating the role of CHN-related disaster vulnerabilities in increasing mental health symptoms among this vulnerable group.

### Parent Mental Health

Parents of youth with CHNs experience unique daily stressors outside of disaster contexts (Isa et al., 2016). These challenges and barriers are likely to exacerbate pre-disaster vulnerabilities and lead to the development of additional vulnerabilities. Given the more hands-on caretaking role that parents often play when caring for youth with CHNs, parents of youth with CHNs are also likely to be exposed to many of the same disaster-related barriers and stressors as their youth (Pickering et al., 2021). Some unique challenges and barriers identified in current literature include extensive pre-disaster planning to accommodate the needs of their youth, evacuation barriers (i.e., severe weather limiting transportation), identifying accessible shelters, and general overwhelm due to the myriad roles that parents of youth with CHNs play in addition to caregiving (Pickering et al., 2021; Ducy & Stough, 2021). These barriers are likely to have some measurable impact on the mental health of caretakers of vulnerable youth with CHNs during disasters.

Parents of youth with CHNs have been identified as experiencing significant distress and anxiety due to navigating disaster while caring for their youth (Ducy & Stough, 2021; Takada, 2013). Parents also tended to report delayed trauma symptom presentation, likely due to emotional suppression, highlighting the focus that these parents placed on the emotional well-being of their children over that of themselves (Ducy & Stough, 2021). Moreover, parents of youth with CHNs during the COVID-19 pandemic were found to exhibit heightened psychopathological symptoms (Mann et al., 2021). These studies provide important insights into the experiences of disaster-affected parents of youth with CHNs; however, it is difficult to generalize the effects of the multi-year pandemic to the effects of short-term disasters and qualitative methodology to the broader population. Additionally, despite the little research on youth with CHNs and their families during disasters, the unique disaster- and CHN-related experiences had by this group likely have a measurable impact on the psychological functioning of both youth with CHNs and their families.

## Current Study

Given the vulnerabilities that youth with CHNs and their families experience during disasters, attention must be given to a better understanding of the unique disaster experiences and how disaster-related and CHN-related factors influence the mental health outcomes of this group. The current study evaluated these experiences and outcomes in youth with CHNs and their parent following hurricane exposure, utilizing parent reports of these experiences. Moreover, such experiences and outcomes were compared with youth without CHNs and their parent. The study aims include:

***Aim 1***. Determine if having a youth with CHNs influences perceptions of hurricane threat and evacuation.

***Aim 2***. Compare mental health outcomes in hurricane-affected youth with CHNs and without CHNs and their parents.

***Aim 3***. Identify factors that impact post-disaster functioning in youth with CHNs and their parent.

## Methods

### Participants

This study recruited parents (*N* = 142) of youth with CHNs (*n* = 48) and without CHNs (*n* = 94) living in hurricane-impacted counties in the state of Florida during Hurricane Ian, a Category 5 hurricane that struck Florida in September 2022. The severe weather generated by the storm led to 156 deaths and almost $113 billion in structural damages (Bucci et al., 2022). Parents reported on their youth’s and their own disaster-related mental health functioning and experiences. See Table 1 for sample demographic information. Table 2 describes the CHNs in the youth in this sample. Within this sample, 34% of youth were identified by their parent as living with a CHN.

**Table 1:** Demographics of study sample

	Parents	Youth
Sex (% female)	73%	47%
Age range	25–64	7–17
Race/Ethnicity		
Non-Hispanic White	77%	72%
Hispanic White	11%	11%
Non-Hispanic Black	8%	8%
Hispanic Black	1%	1%
Asian (Hispanic/Non-Hispanic)	1%	1%
Middle Eastern or North African (Hispanic/Non-Hispanic)	0%	0%
American Indian or Alaska Native	0%	0%
Multiracial/Other (Hispanic/Non-Hispanic)	2%	7%
Parental Education		
Did not complete high school	2%	--
High school degree or equivalent	12%	--
Some college	31%	--
Bachelor’s degree	39%	--
Graduate degree	16%	--
Gross annual income (pre-tax)		
Under $25K	9%	--
$25K - $50K	23%	--
$50K - $75K	21%	--
$75 - $100k	27%	--
Over $100K	20%	--

**Table 2:** Youth with CHNs: Number and type of CHNs in this sample

	%	*n*
***Number of CHNs***		
1 CHN	25%	12
2 CHNs	29%	14
3 CHNs	13%	6
4 CHNs	15%	7
≥5 CHNs	18%	9
***Type of CHN***		
Intellectual or cognitive disability	38%	18
Specific learning disability	42%	20
Autism spectrum disorder	25%	12
Deaf or hard of hearing	8%	4
Blind or low vision	29%	14
Speech or language impairment	17%	8
Developmental delay	25%	12
Conditions present at birth	10%	5
Mobility, movement, or coordination impairment	13%	6
Fine motor impairment	18%	9
Cancer	8%	4
Asthma, allergies, or other respiratory problems	23%	17
Other chronic health condition	31%	15
***Most Common Comorbidities***		
Intellectual or cognitive disability & Specific learning disability	19%	9
Specific learning disability & Respiratory problems	17%	8
Specific learning disability & Other chronic health conditions	17%	8
Intellectual or cognitive disability & Blind or low vision	15%	7
Intellectual or cognitive disability & Other chronic health conditions	15%	7
***Level of Support***		
Requires help with few daily activities	46%	22
Requires help with some daily activities	29%	14
Requires help with most daily activities	19%	9
Requires help with all daily activities	6%	3

*Note*. Percentages reflect those among only youth with CHNs. Some youth have multiple types of CHNs

### Procedure

The present study was approved by the Institutional Review Board at the University of South Dakota. Participants were recruited via social media posts and advertisements, listservs with study information sent to schools, daycares, universities, and other relevant businesses, and flyers publicly posted in target Florida counties. Within these counties, families of youth with CHNs were specifically targeted for recruitment by way of sending study information tailored to youth with CHNs to medical offices and the previously mentioned businesses and organizations. The survey was administered via Qualtrics and took approximately 15 to 20 min. Participants were eligible to participate if they were parents of youth younger than 18 years and lived in an eligible Florida county. Eligible counties were those that were in mandatory evacuation zones during the hurricane. These counties were Lee, Charlotte, Collier, Hardee, Highlands, Sarasota, and Desoto. Eligibility checks included matching the participant’s zip code to those of the target counties, matching the birthdate of the youth to the youth’s reported age, matching reported schools or daycares to those located in target counties, as well as two additional attention checks (see Agley et al., 2022). Upon clicking the survey link, participants reviewed the consent form and were asked to “click” a button to consent to participate. Participants were required to pass four of five eligibility checks and provide a valid mailing address in an eligible Florida county to receive compensation. Participants received a $5 Amazon gift card and an informational coping workbook for their youth for their participation.

### Measures

#### Demographics

Parents provided information regarding sex, age, race, and ethnicity for themselves and their youth. Parental education level and family household income were also collected.

#### Evacuation Experiences and Barriers

Hurricane-related evacuation barriers were evaluated using the Before and After Storm Experiences measure (BASE; La Greca et al., 2019). The BASE is a 15-item measure that assesses exposure to a variety of different disaster-related experiences or barriers as well as the extent of distress that the experience caused. Parents rated these experiences on a 5-point scale (0 = *Event did not occur* to 4 = *Event occurred and was very stressful*). Internal consistency for the current study was *a* = 0.91.

**Perceived Life Threat**. Parent PLT was evaluated using a single item (i.e., “I thought I might die”) included in the Peritraumatic Distress Inventory (PDI; Brunet et al., 2001). Parent reports of youth PLT for themselves and their family members were evaluated using three individual items assessing whether the youth thought they might die or get hurt or whether they thought a family member might be hurt or die (1 = *Yes*, 0 = *No*). Summed total scores were calculated for youth PLT to be used for analyses.

#### Youth Mental Health

**Posttraumatic Stress**. Youth PTS symptoms were evaluated utilizing parent reports from the International Trauma Questionnaire Child and Adolescent Caregiver Version (Cloitre et al., 2018; Lueger-Shuster et al., 2023). The measure uses six items for parents to indicate, over the past month, the frequency with which their youth experiences PTS symptoms using a 5-point Likert scale (0 = *Never* to 4 = *Almost Always*). The total range is 0–24. Internal consistency is good (i.e., *a* = 0.86; Danzi et al., 2024). Internal consistency for the current study was *a* = 0.92. The ITQ summed total scale score was used for analyses.

**Depression and Anxiety**. Youth depressive and anxiety symptoms were evaluated using the parent-report version of the Revised Children’s Anxiety and Depression Scale, 11-item Version (RCADS-11; Radez et al., 2020). Individual summed subscale scores for anxiety and depression were used for analyses. The RCADS-11 anxiety subscale contains six items while the depression subscale contains five items. The total ranges of the anxiety and depression subscales are 0–18 and 0–15, respectively. Parents are asked to indicate how often their youth exhibits a variety of depressive and anxiety symptoms using a 4-point Likert scale (0 = *Never/not at all* to 3 = *Always/a great deal*). Internal consistency is good to excellent (i.e., *a* = 0.82–0.93; Piqueras et al., 2017). Internal consistency for the current study was *a* = 0.93.

#### Parent Mental Health

**Posttraumatic Stress**. Parent PTS symptoms were evaluated using the Posttraumatic Stress Checklist for the DSM-5 (PCL-5; Weathers et al., 2013). The PCL-5 is a 20-item measure that asks parents to indicate how often, in the past month, they were bothered by various PTS symptoms using a 5-point Likert scale (0 = *Not at all* to *4 = Extremely*). The total range is 0–80. Internal consistency is good to excellent (i.e., *a* = 0.83–0.97; Forkus et al., 2023). Internal consistency for the current study was *a* = 0.96. The PCL-5 summed total score was used for analyses. A cut off-score of 31–33 is noted as indicating possible clinically significant symptoms.

**Depression and Anxiety**. Parent depressive and anxiety symptoms were evaluated using the 21-item Depression, Anxiety, and Stress Scale (DASS-21; Lovibond & Lovibond, 1995). The DASS-21 anxiety and depression individual summed subscale scores were used for analyses. The anxiety and depression subscale scores contain seven items with ranges of 0–28, respectively. Parents indicate the extent to which they experienced a variety of depressive and anxiety symptoms on a 4-point Likert scale (0 = *Did not apply to me at all* to 3 = *Applied to me very much*,* or most of the time*). Internal consistency is good to excellent for the depression and anxiety subscales (i.e., *a* = 0.87–0.94; Antony et al., 1998). Internal consistency for the current study was *a* = 0.97. Cut-off scores for the DASS-21 anxiety subscale are as follows: 0–7 (*Normal*), 8–9 (*Mild*), 10–14 (*Moderate*), 15–19 (*Severe*), 20+ (*Extremely severe*). Cut-off scores for the depression subscale are as follows: 0–9 (*Normal*), 10–13 (*Mild*), 14–20 (*Moderate*), 21–27 (*Severe*), 28+ (*Extremely severe*).

#### Complex Health Needs and Related Variables

**Complex Health Needs and Level of Support**. Youth’s specific healthcare-related needs were evaluated by asking their parent to indicate whether their child experienced a variety of CHNs. Categories indicating different healthcare needs or conditions were adapted from those categories used in the Individuals with Disabilities Education Act (2004) and the Centers for Disease Control and Prevention (2020). Health condition categories included intellectual or cognitive disability; specific learning disability; autism spectrum disorder; deaf or hard of hearing; blind or low vision; speech or language impairment; developmental delay; conditions present at birth (e.g., Down’s Syndrome, cystic fibrosis, spina bifida, congenital heart defects); mobility, movement, or coordination impairment; fine motor impairment; cancer; asthma, allergies, or other respiratory problems; chronic health condition (e.g., diabetes, epilepsy); and other health problems. Parents were able to indicate if their youth had multiple of these conditions. The total sum of CHNs identified was used for analyses. The level of support required to care for youth compared to other youth of comparable age was evaluated. Parents indicated the extent to which their youth requires assistance carrying out daily activities due to their medical, behavioral, developmental, or other health condition (1 = *My child requires no help with daily activities* to 5 = *My child requires help with all daily activities*). Regarding youth with CHNs, CHN status (has or does not have a CHN) was determined based on the extent to which the youth with CHNs require help with daily activities. Parents who indicated that their youth *requires no help with daily activities* were categorized as not having CHNs (1 = Has a CHN, 0 = Does not have a CHN).

**Healthcare necessities**. Information regarding whether access to their youth’s healthcare necessities was impacted by the hurricane (1 = *Yes*, 0 = *No*) was evaluated using a single parent-report item. Reliance on healthcare necessities was evaluated using a single parent-report item (1 = *Yes*, 0 = *No*) and covered reliance on the following healthcare necessities: Care from a specialty doctor; prescription medication; eyeglass or vision care; hearing aids or hearing care; mobility aids or devices (e.g., canes, crutches, wheelchairs, or scooters); communication aids or devices (e.g., communication boards); durable medical equipment; dental care; physical therapy; occupational therapy; speech therapy; mental health care or counseling; substance abuse treatment or counseling; home health care; respite care; and genetic counseling.

**Financial Healthcare Concerns**. The degree of concern related to financial hardship in affording their youth’s healthcare necessities was also evaluated (1 = *Not at all concerned* to 4 = *Extremely concerned*).

### Data Analysis

The present study’s analyses were carried out using IBM Statistical Package for the Social Sciences version 29. This study used data collected for youth ages 7 to 17 from a larger dataset that included younger children. Missing data was low (< 1%) and was handled with single mean imputation. Additionally, 26 youths who were identified by their parents as having a health condition but were indicated as not requiring help with daily activities were coded as not having a CHN.

For Aim 1, parent-reported differences in perceived life threat between youth with CHNs and without CHNs, and their parents were evaluated using chi-square tests. The evacuation experiences of families of youth with CHNs were compared with those experiences of families of youth without CHNs. Specifically, a chi-square test was used to compare rates of evacuation between families of youth with CHNs and families of youth without CHNs. A comparison of total evacuation-related barriers faced between families of youth with CHNs and families of youth without CHNs, controlling for time passed since the hurricane, was evaluated using a general linear model with analysis of variance. The same process was used to compare further the unique barriers experienced among families of youth with CHNs and without CHNs. Effect sizes were evaluated using partial eta squared (Valladares-Neto, 2018).

For Aim 2, youth with and without CHNs and their parents were compared on the degree of post-hurricane mental health functioning, using parent reports of functioning. Youth with and without CHNs were compared on PTS, depressive, and anxiety symptom severity, controlling for time since the hurricane. Parents of youth with CHNs and parents of youth without CHNs were compared on PTS, depressive, and anxiety symptom severity, controlling for parent sex and time since the hurricane. Six general linear models with analysis of covariance were utilized to evaluate symptom severity among youth and parents. Effect sizes were evaluated using partial eta squared.

For Aim 3, hierarchical linear regression was used to identify factors that influence PTS, depressive, and anxiety symptom severity in youth with CHNs, using parent reports of these symptoms. Time passed since the hurricane was entered in Step 1, financial healthcare concerns were entered in Step 2, and hurricane-impeded healthcare access was entered in Step 3. This regression model was used with youth PTS, depressive, and anxiety symptoms as the outcome variables. For parents, time passed since the hurricane and sex were entered in Step 1, financial healthcare concerns were entered in Step 2, and hurricane-impeded healthcare access was entered in Step 3. Effect sizes were evaluated using Cohen’s *f* (Valladares-Neto, 2018).

## Results

Univariate and multivariate normality checks revealed non-normality and skewness within primary outcomes variables. Statistical analyses to adjust for this were utilized. Bonferroni correction was used to correct for multiple comparisons. Descriptive statistics (i.e., variable means, standard deviations, and correlations) for target study variables are outlined in Table 3. Most study variables were strongly to very strongly correlated (all *p*s < 0.01). Parent-reported youth depressive symptom severity was moderately correlated with parent PTS symptom severity (*p* < .01). No demographic differences between youth with and without CHNs and their parents were observed for youth and parent age, SES, income, and race and ethnicity. No significant differences were observed for youth sex. Parents of youth without CHNs were significantly more likely to be female (χ^2^(1, *N* = 142) = 13.46, *p* < .001). Thus, parent sex and the time since the hurricane were used as covariates in analyses.

**Table 3:** Means, standard deviations, and correlations for primary variables of interest

	M (SD)	1	2	3	4	5
1. Youth PTS	4.87 (*5.53*)	--				
2. Youth Depressive	2.56 (*3.05*)	0.68**	--			
3. Youth Anxiety	4.07 (*3.85*)	0.77**	0.82**	--		
4. Parent PTS	22.27 (*18.91*)	0.80**	0.59**	0.69**	--	
5. Parent Depressive	10.59 (*11.39*)	0.72**	0.64**	0.69**	0.78**	--
6. Parent Anxiety	12.27 (*12.18*)	0.77**	0.63**	0.72**	0.83**	0.88**

**p < .01

Sample means indicated low severity for PTS, depressive, and anxiety symptomatology in the overall youth sample. Sample means for youth PTS, depressive, and anxiety symptom severity were in the lowest quartile of their respective ranges. Among parent PTS symptom severity in the sample, 66% of parents fell below the indicated cut-off scores for clinically significant (i.e., 31–33). Regarding parental depressive symptoms, symptom severity fell within the following ranges: *Normal* (54%), *Mild* (10%), *Moderate* (12%), *Severe* (14%), and *Extremely severe* (8%). Parent anxiety symptom severity fell within the following ranges: *Normal* (48%), *Mild* (4%), *Moderate* (9%), *Severe* (9%), and *Extremely severe* (10%).

### Aim 1: Determine if Having a Youth with CHNs Influences Perceptions of Hurricane Threat and Evacuation

For youth with CHNs, parent reports indicated that 46% thought that they might die, 65% thought that they might be injured, and 63% thought that a family member might be injured or die due to the hurricane. For youth without CHNs, parent reports indicated that 26% of youth thought that they might die, 48% thought they might be injured, and 49% thought that a family member might be injured or die. Youth with CHNs exhibited heightened perception that they might die from the hurricane (χ^2^(1, *N* = 142) = 5.98, *p* = .014). There were no differences between youth with versus without CHNs for the perception that they might be injured (χ^2^(1, *N* = 142) = 3.57, *p* = .059) or that a family member might be hurt badly or die (χ^2^(1, *N* = 142) = 2.35, *p* = .125).

For parents of youth with CHNs, 58% of parents thought they might die. For parents of youth without CHNs, 48% of parents thought they might die. There were no differences observed between parents of youth with CHNs and parents of youth without CHNs in perceived life threat (χ^2^(1, *N* = 142) = 1.39, *p* = .238).

Among the entire sample, 51% of families evacuated during the hurricane: 63% of families of youth with CHNs evacuated, whereas only 45% of families of youth without CHNs evacuated. Families with youth with CHNs evacuated at a greater rate than families of youth without CHNs (χ^2^(1, *N* = 142) = 4.04, *p* = .045). Additionally, families of youth with CHNs experienced greater evacuation barriers (*M* = 8.46, *SD* = 0.66) compared to families without a youth with CHNs (*M* = 6.03, *SD* = 0.47), *F(*1, 139) = 8.85, *p* = .003, after controlling for time passed since the hurricane. Effect sizes were small (*ηp*^2^ = 0.001–0.04) regarding differences in evacuation experiences; however, the effect size was medium (*ηp*^2^ = 0.09) for car troubles. Families of youth with CHNs were determined to have significantly greater evacuation barriers and related stress compared to families of youth without CHNs for several identified barriers. See Table 4 for specific evacuation barriers experienced by families of youth with CHNs and families of youth without CHNs and whether these experiences were different between groups.

**Table 4:** Comparison of barriers to evacuation between families of youth with CHNs and families of youth without CHNs

	CHNs	No CHNs	
	*M* (*SD*)	*M* (*SD*)	*F*(1, 2)
Witnessing arguments/physical fights	1.33 (*1.45*)	0.90 (*1.41*)	0.56*
Disagreements with family about what to bring with or leave behind	1.54 (*1.38*)	0.96 (*1.29*)	3.76*
Trouble finding transportation	1.52 (*1.50*)	0.87 (*1.37*)	6.80**
Trouble finding a place to stay	1.58 (*1.60*)	0.96 (*1.42*)	5.01**
Car breaking down or car accident	1.27 (*1.53*)	0.44 (*1.04*)	8.51***
Trouble finding/getting into a shelter	1.46 (*1.53*)	0.79 (*1.37*)	6.42**
Getting stuck somewhere	1.29 (*1.40*)	0.63 (*1.20*)	5.62**
Separation from a key family member	1.31 (*1.42*)	0.69 (*1.33*)	5.64**
Changing plans due to cancelled flights or car problems	1.08 (*1.50*)	0.52 (*1.12*)	5.93**
Trouble getting gasoline	2.13 (*1.39*)	2.10 (*1.46*)	1.04^*ns*^
Stuck in traffic/travel delays	1.63 (*1.45*)	1.24 (*1.43*)	1.13^*ns*^
Cancelled airline flight	0.83 (*1.29*)	0.52 (*1.15*)	2.37^*ns*^
Changing plans due to changes in the Hurricane’s projected path	0.87 (*1.45*)	0.87 (*1.35*)	2.35^*ns*^
Disagreements with family about whether to evacuate or not	1.85 (*1.49*)	1.73 (*1.46*)	.143^*ns*^
Trouble deciding whether to stay or leave	2.06 (*1.42*)	2.01 (*1.44*)	.150^*ns*^

*p < .05, **p < .01, ***p < .001, *ns* = not significant

### Aim 2: Compare Mental Health Outcomes in Hurricane-affected Youth with CHNs and without CHNs and their Parent

Youth with CHNs and youth without CHNs were compared on PTS, depressive, and anxiety symptom severity utilizing parent reports of symptoms (Table 5). Overall, youth with CHNs exhibited greater PTS, depressive, and anxiety symptom severity compared to youth without CHNs when controlling for time passed since the end of the hurricane. A medium effect was observed for youth PTS (*ηp*^2^ = 0.06) and anxiety symptom severity (*ηp*^2^ = 0.09), while a small effect was observed for youth depressive symptom severity (*ηp*^2^ = 0.04). Parents of youth with CHNs and parents of youth without CHNs were compared on PTS, depressive, and anxiety symptom severity (Table 5). Parents of youth with CHNs exhibited greater PTS, depressive, and anxiety symptom severity compared to parents of youth without CHNs when controlling for parent sex and time passed since the end of the hurricane. Small effects were observed for parent PTS, depressive, and anxiety symptom severity (*ηp*^2^ = 0.01–0.03).

**Table 5:** Mental health symptom severity in youth with CHNs and without CHNs and their parents

	CHNs	No CHNs	
	*M* (*SD*)	*M* (*SD*)	
**Youth**			
PTS	6.98 (*6.13*)	3.80 (*4.89*)	*F*(1, 2) = 13.16**
Depressive	3.48 (*3.33*)	2.10 (*2.80*)	*F*(1, 2) = 6.52**
Anxiety	5.79 (*4.17*)	3.19 (*3.36*)	*F*(1, 2) = 10.38***
**Parent**			
PTS	28.54 (*20.23*)	19.06 (*17.45*)	*F*(1, 3) = 15.83***
Depressive	15.04 (*12.10*)	8.32 (*10.36*)	*F*(1, 3) = 14.41***
Anxiety	17.38 (*13.57*)	9.66 (*10.56*)	*F*(1, 3) = 17.23***

*p < .05, **p < .01, ***p < .001, *ns* = not significant

### Aim 3: Identify Factors that Impact Post-disaster Functioning in Youth with CHNs and their Parent

Among families of youth with CHNs, 94% of families reported experiencing some degree of concern related to affording their youth’s healthcare needs. Additionally, 75% of youth with CHNs were indicated by parents to utilize specific healthcare necessities to support their functioning. Moreover, 75% of youth with CHNs had hurricane-impeded access to healthcare necessities.

#### Youth Mental Health

**Posttraumatic Stress Symptoms**. Youth PTS symptom severity was not associated with time passed since the hurricane (Table 6). Financial healthcare concern was not associated with youth PTS symptom severity. Lastly, greater hurricane-impeded access to healthcare was associated with greater youth PTS symptom severity, accounting for a large effect (*f*^2^ = 0.90).

**Table 6:** Factors associated with the mental health of youth with CHNs

Variable	B	SE B	B	t	*p*	*R* ^2^
**PTS**						
Time						0.05^*ns*^
Days Since the Hurricane	− 0.03	0.02	− 0.23	1.61	0.115	
Healthcare Finances						0.09^*ns*^
Level of Concern	1.31	0.95	0.20	1.38	0.174	
Hurricane-Impeded Healthcare						0.05***
Impeded Healthcare Status	8.51	1.60	0.61	5.33	< 0.001	
**Depressive**						
Time						0.05^*ns*^
Days Since the Hurricane	− 0.01	0.01	− 0.21	− 1.45	0.147	
Healthcare Finances						0.07^*ns*^
Level of Concern	0.60	0.52	0.17	1.16	0.253	
Hurricane-Impeded Healthcare						0.23**
Impeded Healthcare Status	3.11	1.02	0.41	3.04	0.004	
**Anxiety**						
Time						0.01^*ns*^
Days Since the Hurricane	− 0.01	0.01	− 0.10	− 0.70	0.485	
Healthcare Finances						0.06^*ns*^
Level of Concern	0.98	0.66	0.22	1.48	0.145	
Hurricane-Impeded Healthcare						0.27^*ns*^
Impeded Healthcare Status	24.46	1.25	0.47	3.56	< 0.001	

Note. ^a^ 0 = Male, 1 = Female. Significance of overall model *F*-Statistic: **p < .01, ***p < .001, *ns* = not significant

**Depressive Symptoms**. Youth depressive symptom severity was not associated with time passed since the hurricane (Table 6). Financial healthcare concern was not associated with youth depressive symptom severity. Greater hurricane-impeded access to healthcare was associated with greater youth depressive symptom severity, accounting for a large effect size (*f*^2^ = 0.55).

**Anxiety Symptoms**. Youth anxiety symptom severity was not associated with time passed since the hurricane (Table 6). Financial healthcare concern was not associated with youth anxiety symptom severity. Greater hurricane-impeded access to healthcare was associated with greater youth anxiety, accounting for a large effect size (*f*^2^ = 0.61).

#### Parent Mental Health

**Posttraumatic Stress Symptoms**. Parent PTS symptom severity was associated with parent sex, such that being female was associated with greater PTS symptom severity (Table 7). Parent PTS was not associated with time passed since the hurricane. Financial healthcare concerns were not associated with parent PTS symptom severity. Greater hurricane-impeded access to healthcare was associated with greater parent PTS symptom severity, accounting for a large effect size (*f*^2^ = 0.99).

**Table 7:** Factors associated with the mental health of parents of youth with CHNs

Variable	B	SE B	B	t	*p*	*R* ^2^
**PTS**						
Covariates						0.36***
Sex^a^	− 23.94	4.93	− 0.60	− 4.86	< 0.001	
Days Since the Hurricane	− 0.01	0.05	− 0.02	− 0.13	0.897	
Healthcare Finances						0.36***
Level of Concern	0.03	2.84	0.00	0.01	0.991	
Hurricane-Impeded Healthcare						0.50***
Impeded Healthcare Status	20.21	5.93	0.44	3.41	0.001	
**Depressive**						
Covariates						0.41***
Sex^a^	− 15.81	2.83	− 0.66	− 5.59	< 0.001	
Days Since the Hurricane	0.04	0.03	0.17	1.45	0.154	
Healthcare Finances						0.43***
Level of Concern	1.79	1.61	0.14	1.11	0.273	
Hurricane-Impeded Healthcare						0.44***
Impeded Healthcare Status	3.72	3.74	0.13	0.99	0.326	
**Anxiety**						
Covariates						0.40***
Sex^a^	− 17.36	23.20	− 0.65	− 65.43	< 0.001	
Days Since the Hurricane	0.02	0.03	0.07	0.59	0.561	
Healthcare Finances						0.41***
Level of Concern	1.25	1.84	0.09	0.68	0.499	
Hurricane-Impeded Healthcare						0.48***
Impeded Healthcare Status	9.69	4.06	0.31	2.39	0.021	
Note. ^a^ 0 = Male, 1 = Female. Significance of overall model *F*-Statistic: ***p < .001.						

**Depressive Symptoms**. Parent depressive symptom severity was associated with parent sex but not time passed since the hurricane (Table 7). Specifically, being female was associated with greater depressive symptom severity. Financial healthcare concerns were not associated with parent depressive symptomatology. Hurricane-impeded access to healthcare was also not associated with parent depressive symptomatology, accounting for a large effect size (*f*^2^ = 0.88).

**Anxiety Symptoms**. Parent anxiety symptom severity was associated with parent sex, such that being female was associated with greater anxiety symptom severity (Table 7). There was no association found with time passed since the hurricane. Financial healthcare concerns were not associated with parent anxiety symptom severity. Finally, greater hurricane-impeded access to healthcare was associated with greater parent anxiety symptom severity, accounting for a large effect size (*f*^2^ = 0.95).

## Discussion

Youth with CHNs and their parents experience significant day-to-day vulnerabilities that complicate functioning and impact mental health (Goudie et al., 2014; Werner & Shulman, 2013; Berg et al., 2015), and exposure to natural disasters further magnifies these vulnerabilities (Rath et al., 2007; Ducy & Stough, 2021). As such, youth with CHNs and their parents are at risk of adverse psychological functioning following disasters, and these vulnerabilities warrant further attention. The purpose of this study was to evaluate disaster-related experiences, perceptions, and mental health functioning of disaster-exposed youth with CHNs and their parents. Further, to our knowledge, this is the first study to specifically evaluate how specific CHN-related factors (i.e., financial healthcare concerns and whether the hurricane impacted the youth’s healthcare necessities) impact the mental health of youth with CHNs and their parents.

### Evacuation Experiences and Perceptions of Hurricane Threat among Youth with CHNs and Their Families

Results from the current study revealed that youth with CHNs were observed to exhibit a heightened perception of the threat of dying during the hurricane but not being injured or a family member being injured or killed, per parent report. While no research to date has specifically evaluated perceived life threat in youth with CHNs exposed to disasters, the findings of the present study are not surprising given the strong link between PLT and PTS (McDonald et al., 2019). Regarding youth PLT, due to the high parent-indicated reliance of youth with CHNs on medical necessities as well as their parents for carrying out daily tasks and ensuring their health is maintained, it may be that youth with CHNs are more sensitive to the possibility of a disaster impacting their access to the resources that support their survival compared to youth without CHNs (Peek & Stough, 2010; Ducy & Stough, 2021; Stough et al., 2017). No differences were observed in PLT between parents of youth with CHNs and parents of youth without CHNs. Such a finding may have occurred for several reasons. It may be that parents in this sample mirrored those parents identified as being too preoccupied with ensuring the well-being of their youth that they “masked” their fears or failed to attend to their own emotions (Ducy & Stough, 2021). Alternatively, resiliency factors not identified in the current study may have been protective for parents in this group. Such findings highlight the need for future research into risk and resiliency factors for youth with CHNs and their parents who are exposed to disasters.

Regarding evacuation experiences, parents indicated that youth with CHNs and their families were more likely to evacuate from their homes compared to families of youth without CHNs. Additionally, during evacuation, families of youth with CHNs experienced greater barriers and greater stress during this process compared to families of youth without CHNs even after controlling for time passed since the hurricane. Thus, families of youth with CHNs had more difficulty evacuating but were still more likely to do it anyway; this finding may be linked to the higher perception of threat identified in youth with CHNs. While the rates of evacuation barriers experienced by youth with CHNs and their families are consistent with the literature, the finding that families of youth with CHNs evacuated at a greater rate than families of youth without CHNs adds to prior research that suggests provides contradictory findings regarding evacuation rates among this group (Peek & Stough, 2010; Ducy & Stough, 2021). The findings observed in the present study may be due to several factors not specifically evaluated, such as unique aspects of hurricanes related to other forms of natural disasters or unique CHN-related factors that differ from youth to youth. Additionally, it is important to note that time passed since the hurricane did not have a measurable impact on evacuation perceptions.

The current study also revealed that families of youth with CHNs were more likely to experience transportation-related barriers, trouble finding shelters, becoming stranded during evacuation, separation of family members, changing evacuation plans, and witnessing interpersonal disagreements compared to families of youth without CHNs. These findings mirror those barriers observed in other studies; however, these findings highlight additional vulnerabilities (e.g., interpersonal disagreements) that may be uniquely experienced by families of youth with CHNs and warrant further consideration (Peek & Stough, 2010; Ducy & Stough, 2021; Stough et al., 2017).

### Mental Health in Youth with CHNs and Their Parents after a Hurricane

As expected, youth with CHNs exhibited greater PTS, depressive, and anxiety symptom severity compared to youth without CHNs. This was true even after controlling for the time that had passed since the hurricane. Parents of youth with CHNs also exhibited greater PTS, depressive, and anxiety symptom severity compared to parents of youth without CHNs, after controlling for the time that had passed since the hurricane and parent sex. These findings are consistent with and support the small yet growing literature highlighting these groups’ poorer mental health functioning, specifically those exposed to disasters (Rath et al., 2007; Ducy & Stough, 2021; Takada, 2013; Mehtar & Mukaddes, 2011). Additionally, it is likely that the unique CHN-related factors and disaster experiences faced by families of youth with CHNs drive the heightened discrepancies in symptom severity between these groups. These findings highlight the significant physical and psychological vulnerabilities of disaster-exposed families of youth with CHNs. Moreover, these findings, coupled with the limited integration of the unique needs of this group in disaster management and response, highlight the need for continued development of these policies to better support vulnerable youth and families.

### Factors Associated with Mental Health Functioning in Youth with CHNs Post-Disaster

Among youth with CHNs, time that had passed since the hurricane was not associated with any mental health outcomes, per parent reports of youth mental health functioning. Due to the fluctuations in mental health symptoms following trauma exposure, this variable typically emerges as a relevant predictor of psychological distress (Tay et al., 2017). However, other factors may be more salient in youth with CHNs within this sample.

Financial healthcare concern was not associated with youth PTS, depressive, or anxiety symptom severity among youth with CHNs, when controlling for time. Moreover, greater hurricane-impeded access to healthcare was associated with greater youth PTS, depressive, and anxiety symptom severity. The insignificant associations between financial healthcare concerns and youth mental health symptom severity were surprising given the literature that suggests that youth are often sensitive to parental financial stress (Zimmer-Gembeck et al., 2022). However, as previously noted, factors directly impacting youth well-being (e.g., hurricane-impeded access to healthcare necessities) were more salient in impacting mental health among this medically vulnerable population. Nonetheless, these findings provide critical insights into the extent to which vulnerability factors impact mental health functioning among this group. Indeed, these findings highlight the significant vulnerabilities faced by youth with CHNs during disasters, including impacted access to critical, often life sustaining, necessities.

### Factors Associated with Mental Health Functioning in Parents of Youth with CHNs

For parents of youth with CHNs, females had greater PTS, depressive, and anxiety symptom severity. This is consistent with current literature discussing the influence of sex on post-disaster symptom severity, such that males generally have lower post-disaster distress than females (Steinert et al., 2015). Additionally, the amount of time that had passed since the hurricane was not associated with parent PTS, depressive, or anxiety symptom severity, suggesting no influence of time on perceptions of mental health symptoms. Further research is needed to better understand these issues.

Financial healthcare concerns were not associated with parent PTS, depressive, or anxiety symptom severity. Moreover, greater hurricane-impeded access to healthcare was associated with greater parent PTS and anxiety symptom severity but not parent depressive symptom severity. The lack of association between financial healthcare concerns and parents’ mental health symptom severity was unexpected. It was expected that, because parents are the main financial managers within the family and finances among families of youth with CHNs are often more complex due to increased medical expenses, greater concern for affording their youth’s healthcare necessities would have a notable impact on parents’ mental health. It may be the case for parents among this sample that factors more directly affecting their youth’s well-being (i.e., hurricane-impeded access to healthcare) were more salient in impacting parents’ mental health compared to financial concerns. Similarly, parents may have suppressed their adverse emotional reactions to factors more directly impacting them (e.g., financial healthcare concerns to support the well-being of their youth (Ducy & Stough, 2021).

The lack of association between financial healthcare concerns and hurricane-impeded access to healthcare on parent depressive symptom severity was also unexpected. It may be that ongoing difficulties related to post-disaster recovery efforts played a greater role in parent PTS and anxiety symptom severity compared to depressive symptom severity. More research is needed to better understand the unique ways in which caring for a youth with CHNs impacted the post-disaster functioning of hurricane-affected parents.

Indeed, results from the present study highlight how unique CHN-related (i.e., financial healthcare concerns) and disaster-driven (i.e., hurricane-impeded access to healthcare needs) might adversely impact post-disaster functioning among youth with CHNs and their parents. The present study is the first to quantitatively evaluate the unique impact of such vulnerabilities on these groups’ mental health outcomes among youth with a variety of CHNs. In general, findings from this study support current research on and accounts of disaster-affected parents of youth with CHNs regarding their own and their youth’s mental health and how such vulnerabilities uniquely impacted their mental health (Ducy & Stough, 2021; Takada, 2013). The findings from this study may provide an additional framework for future evaluation of parents of youth with CHNs exposed to disasters.

### Limitations and Future Directions

The limitations of the current study are important to note. First, this study only evaluated parent-report data of youth and parent perceptions and functioning at one time point, which limits the ability to generalize these findings over time. Future studies should attempt to evaluate the mental health of this group and possible protective factors at multiple time points post-disaster. Relatedly, evaluating and identifying protective factors at other socio-ecological levels (e.g., peer relationships, family relationships, neighborhood characteristics including community support and disaster-response policy) should be evaluated in future research, as these factors have been indicated to influence resiliency in disaster-exposed populations (Gim & Shin, 2022).

Additionally, due to the high correlations between parent-reported youth and parent mental health symptom severity, interpretation of youth mental health symptom severity is limited (Makol et al., 2019). It may be that, when parents reported on their youth’s mental health, their mental health symptoms were reflected in their youth’s scores. As such, future researchers should seek to obtain both youth and parent report data to evaluate youth mental health symptoms more accurately, reduce the impact of shared method variance, and ensure the validity of youth mental health symptoms. Further, our sample of youth with CHNs was highly heterogeneous, and the type of CHN reported revealed high comorbidity. This high heterogeneity poses limitations when generalizing these results to youth with only a single type of CHN, as the degree of severity and impairment to functioning likely highly differs between CHN types. Additionally, the current study did not specifically evaluate whether disaster-related perceptions, experiences, and psychological functioning differed in youth with CHNs and their parent by type of CHN, as the sample sizes within CHN groups were small. Future researchers should seek to recruit larger samples of youth with CHNs to better evaluate differences in mental health outcomes among different CHN types and assess these differences to provide a comprehensive understanding of the interplay of CHNs and natural disasters and understand the statistical limitations associated with comparing groups using smaller sample sizes. Further, while our sample was predominantly White, the sample reflected the demographics in the counties where participants were recruited. Moreover, while a small percentage of our sample identified as Hispanic/Latino, other ethnically diverse groups were not identified. Similar limitations are observed for diverse gender identities. Future researchers should attempt to recruit racially, ethnically, and gender-diverse participants to further evaluate the unique disaster-related experiences faced by diverse individuals and families (Buchanan et al., 2021; American Psychological Association, 2015).

Future directions regarding evaluating the experiences of families of youth with CHNs may continue to evaluate specific CHN-related and disaster-driven vulnerabilities faced by these groups and how these vulnerabilities influence the mental health of youth and their caretakers. For instance, the present study evaluated the impact of financial healthcare concerns and whether access to their youth’s healthcare needs was impacted by the hurricane. Future researchers may seek to evaluate other specific vulnerabilities (e.g., navigating inaccessible disaster shelters) and how these vulnerabilities identified throughout current literature play a role in adverse responses to disasters for youth with CHNs and their parents (Peek & Stough, 2010; Ducy & Stough, 2021; Stough et al., 2017). Similarly, factors present that may serve as protective or resiliency factors should also be assessed to enhance available resources that may support psychological recovery.

### Clinical Implications

The results of this study have significant clinical implications for this population. First, this study supported the notion that not only are youth with CHNs and their parents physically vulnerable during disasters, but they are also susceptible to poor psychological functioning following disasters. Moreover, the mental health of this group is further complicated by their healthcare needs, highlighting this unique vulnerability. Such information is critical in identifying groups most needing services following natural disasters. Additionally, the results of this study highlight the need for services that are sensitive to the unique experiences of youth with CHNs and their caregivers. By seeking to include youth with CHNs, their parents, and the unique CHN-related needs and experiences in critical disaster planning stages, resiliency and physical well-being may be supported and enhanced for this population.

## Data Availability

The data supporting this study’s findings are available upon request from the corresponding author (J.K.)
